# An Anti-Noise Fast Circle Detection Method Using Five-Quadrant Segmentation

**DOI:** 10.3390/s23052732

**Published:** 2023-03-02

**Authors:** Yun Ou, Honggui Deng, Yang Liu, Zeyu Zhang, Xin Lan

**Affiliations:** School of Physics and Electronics, Central South University, Lushan South Road, Changsha 410083, China

**Keywords:** circle detection, anti-noise, five-quadrant segmentation

## Abstract

Extracting circle information from images has always been a basic problem in computer vision. Common circle detection algorithms have some defects, such as poor noise resistance and slow computation speed. In this paper, we propose an anti-noise fast circle detection algorithm. In order to improve the anti-noise of the algorithm, we first perform curve thinning and connection on the image after edge extraction, then suppress noise interference by the irregularity of noise edges and extract circular arcs by directional filtering. In order to reduce the invalid fitting and speed up the running speed, we propose a circle fitting algorithm with five quadrants, and improve the efficiency of the algorithm by the idea of “divide and conquer”. We compare the algorithm with RCD, CACD, WANG and AS on two open datasets. The results show that we have the best performance under noise while keeping the speed of the algorithm.

## 1. Introduction

Images are the basis of human understanding of the world. Accurate and stable acquisition of image information has always been one of the basic problems of computer vision. As one of the most common graphics in daily life, circles often contain a lot of information. How to detect circles in images quickly and stably has always been a difficult and important point in computer vision. At present, circle detection is widely used in borehole detection [1], glass bottle mouth detection [2], solder joint detection [3], medical object detection [4], radar image detection [5], accurate detection of Aeronautical bearings [6] and other areas.

Hough transform [7] (HT) is the most classical circle detection method. Its basic idea is to convert the original information on the image to the parameter space for voting, and then find local peaks in the parameter space to determine the circular parameter information. This method has strong robustness, but the algorithm needs to vote on any three points in the parameter space, and the time complexity and space complexity are very high. In order to solve the shortcomings of the HT algorithm, scholars continue to propose new algorithms, which can be roughly divided into the following two categories:

The first is a circle detection algorithm based on random sampling, which usually obtains the parameters of candidate circles by random sampling. It only needs one correct sampling to find the true circle, and it is often robust. However, this kind of algorithm needs massive iteration and takes a lot of time. The random Hough transform (RHT) algorithm proposed by Xu et al. [8] is a classic example, which finds the true circle in the image by randomly selecting three points and voting in the parameter space. RHT saves some time compared with the HT algorithm, but the space requirements are still very high. In order to solve this defect, Chen et al. [9] proposed a random circle detection algorithm (RCD). The algorithm samples one more point than the RHT algorithm and replaces the parameter link list [10] in the HT and RHT algorithms with the fourth point. RCD reduces the consumption of space, but the probability of random sampling of four points on the same circle is very low, that is, the probability of correct sampling is very low. To solve this problem, Jiang et al. [11] proposed a sampling optimization algorithm based on differential regions. After each sampling, the sampling result is judged once. If the number of points on the candidate circle reaches a certain number, several samples will be extracted from the difference region. This method improves the probability of correct sampling to some extent, but the accuracy of the algorithm decreases rapidly when random noise occurs in the image. In contrast with Jiang’s method, Wang et al. [12] proposed a method of subpixel random sampling. Specifically, they just randomize one point, and then find other points on the circle through the gradient rule of the edge. The algorithm is optimized in time, but the performance of the algorithm degrades rapidly when noise occurs in the image.

Another is contour-based circle detection algorithm, which usually connects edge points into curves and then obtains circle parameters by analyzing the information of the curves. Such methods tend to detect more accurately and run faster. However, these algorithms rely on the results of edge extraction and often have poor performance for images with a large number of edge curves crossing each other or with noise. Le et al. [13] used a segment detector [14] to extract circular curves, and then performed least-squares fitting to obtain circular parameters. Although this method achieves good performance, there are also problems with useless least squares fitting and redundant calculation caused by straight lines [15]. Different from Le’s method, Yao et al. [16] proposed a circle detection algorithm based on the curvature of curves, which estimates circle parameters by curvature, and filters the radius iteratively in layers. However, the radius layer of the algorithm needs to be preset when the size of the image increases, and the efficiency of the algorithm decreases rapidly [17]. Liu et al. [15] proposed an edge thinning and corner detection algorithm, which increased the accuracy of arc segmentation and improved the speed of operation, but the performance of the algorithm is not good in noisy images. Zhao et al. [18] proposed a circle detection algorithm based on an inner tangent triangle, which improved the accuracy of circle detection by using the area calculation. However, compared with the algorithms of Liu and Le, the running time of the algorithm is not ideal. Ou et al. [17] proposed a circle detection algorithm based on information compression, which compresses points on arcs with the same geometric properties into one point, and then uses points instead of curves to fit circle parameters. However, this method relies on the results of edge detection and does not work well under the interference of higher levels of Gaussian noise.

In general, the random sampling-based circle detection algorithm is time-consuming, while the contour-based circle detection algorithm relies on the edge detection results and is sensitive to noise. Aiming at these two problems, this paper proposes an anti-noise fast circle detection method using five-quadrant segmentation. While improving the accuracy and speed of the circle detection algorithm, the algorithm can maintain good performance under the interference of Gaussian noise. We first perform curve thinning and connection on the image after edge extraction, then suppress noise interference by the irregularity of noise edges and propose circular arcs by directional filtering, then we classify and fit the circular arcs using the five-quadrant segmentation method, and the final true circle is obtained according to the coincidence relationship between the circle and the arc and the average sampling result. The experimental results show that our method has the advantages of high speed, strong noise immunity and high accuracy.

The contributions of this paper are as follows:

(1) We propose a noise suppression algorithm and an arc segmentation algorithm, which can suppress the interference of noise while preserving the arc.
(2) We use a five-quadrant segmentation least-squares circle fitting algorithm, which avoids many invalid fittings, and is verified by the coincidence ratio.

The structure of the rest of this paper is as follows: Section 2 is the Materials and Methods, which details the principle of our circle detection algorithm. Section 3 is the proposed circle detection algorithm, which mainly introduces the process of the algorithm. Section 4 is the Results, mainly compared with other algorithms on the public dataset, and the indicators for comparison include Precision, Recall, F-measure and Time. The Section 5 is the Discussion, which mainly summarizes the full text and proposes future research directions.

## 2. Materials and Methods

Our method mainly includes three steps of image preprocessing, noise suppression and arc segmentation, and circle detection. The function of image preprocessing is mainly to extract edge points and connect them into arcs. The function of suppressing noise interference and arc segmentation is to exclude noise curves and divide the curves into arc segments. The process of circle detection is to fit arcs and find the true circle.

### 2.1. Image Preprocessing

#### 2.1.1. Edge Extraction

First we use a 5 × 5 median filter to reduce interference. Then the adaptive canny [19] edge extraction algorithm provided by Matlab is used to obtain edge point information, which is also the choice of most algorithms [15,17,18].

#### 2.1.2. Contour Refinement and Curve Extraction

**Figure 1:**
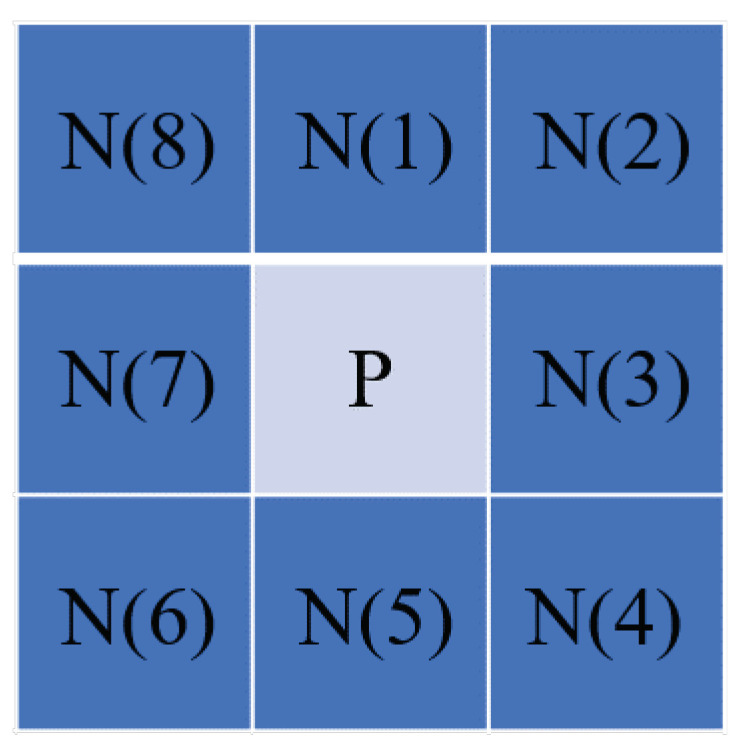
Location of N(1–8) around P.

The thickness of the curve affects arc segmentation and may lead to duplicate detections. First, the method in the literature [15] is used to extract the refined contour. When point P meets any of the following conditions, it is removed from the image.

(1) $$\begin{array}{c} N(1)N(3)+N(3)N(5)+N(5)N(7)+N(7)N(1)=\mathrm{true}\;\&\;N(P)=2 \end{array}$$

(2) $$\begin{array}{c} N(N(1)+N(5))(N(3)+N(7))=\mathrm{true}\;\&\;N(P)>2 \end{array}$$

(3) $$\begin{array}{c} N(1)N(4)N(6)+N(2)N(5)N(8)N(2)N(4)N(7)+N(3)N(6)N(8)=\mathrm{true} \end{array}$$

(4) $$\begin{array}{c} \mathrm{Nc}(P)>2 \end{array}$$

where N(P) is the number of foreground points in the eight-neighbor of P, N(1–8) is the eight-neighbor of P, as shown in Figure 1, and Nc(P) is the number of foreground pixels in N(2), N(4), N(6), and N(8). We specified that the contour curve pixel logical value is 1 (true), and the background pixel logical value is 0 (false). Conditions (1)–(4) should be applied in a logical order. The part without pixels is treated as background pixels when P is on the image’s border.

Then connect all adjacent edge points into curves. Note that curves with a length less than 15 will be deleted. The final extracted curve format is as follows:

(5) $$\begin{array}{c} L=\left(\begin{array}{ccccc} X_{1} & X_{2} & X_{3} & \cdots \cdots & X_{n}\\ Y_{1} & Y_{2} & Y_{3} & \cdots \cdots & Y_{n} \end{array}\right) \end{array}$$

### 2.2. Suppression of Noise Interference and Arc Segmentation

**Figure 2:**
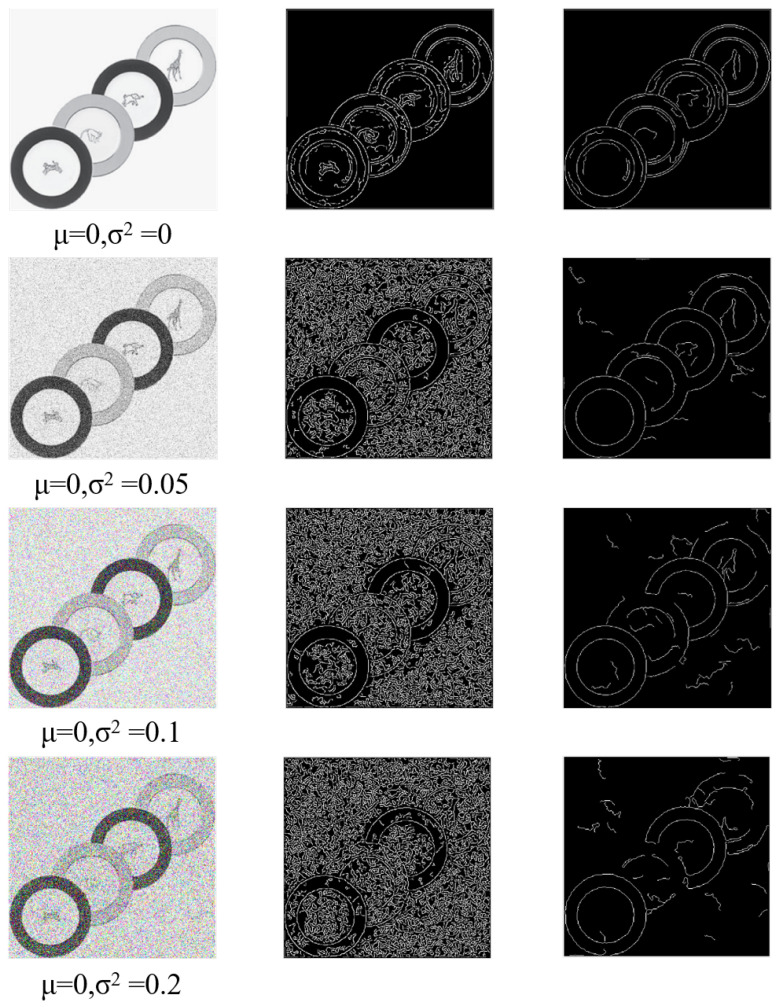
The results of our algorithm at different noise levels.

Under noise interference, the canny algorithm will detect many interference edges. Figure 2 shows the impact of Gaussian noise with different variances on the image and edge detection.

#### 2.2.1. Suppression of Noise Interference

First, we perform a least squares arc fitting on curve L (Equation (5)) to approximate the degree of bending of the arc, and delete the satisfied r<15||r>max(m,n) curve, m, n representing the size of the picture, and r is the radius of the circle fitted by the least squares arc fitting. The Figure 2 shows the effect of our method on pictures at different noise levels.

#### 2.2.2. Arc Segmentation

**Figure 3:**
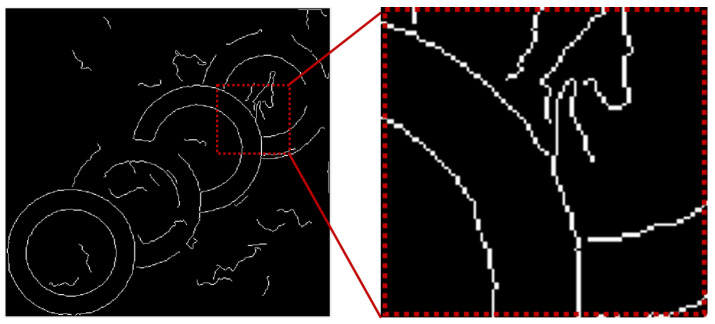
Intermediate results of the algorithm.

Although the curve obtained by the above steps reduces the interference of a part of the noise, there are still some curves that do not belong to the same circle connected together, which leads to an error in fitting, as shown in the Figure 3. Thus, we cut the curve into an arc in the direction. When the Manhattan distance from the ith point to the endpoint is greater than the i${}^{-1}$th point, they are considered to be in the same direction, otherwise the error is recorded with parameters. When the number of recorded errors reaches three, the arc will be taken and the next selected endpoint will be reset. After all the curves are split, the arcs whose curve length is less than 15 will be deleted.

### 2.3. Circle Detection

#### 2.3.1. Five-Quadrant Division Method

In order to reduce the ineffective arc fitting and speed up the fitting, we use the five-quadrant segmentation method to classify the arc. The entire image region is divided into five regions, with the image center as the axis origin. For the arc $L_{i}$, it is fitted to the least squares circle to obtain the circle parameter [X, Y, R], and divides the arc into the corresponding quadrant according to the coordinates of the center of the circle. It should be noted that if the distance between the center coordinate and the coordinate axis is less than r × 0.25, it should not only be placed in the original quadrant, but also in the fifth quadrant. Additionally, the position of the two ends of the arc relative to the center of the circle is calculated, where cx, cy are the endpoint coordinates. and the arc that satisfies condition (6) should be deleted.

(6) $$\begin{array}{c} \left|\frac{\mathrm{angle}2-\mathrm{angle}1}{360}\times 2\times \mathrm{pi}\times R-\mathrm{size}\left(L_{i}\right)\right|>0.25\times R \end{array}$$

where angle1, angle2 are the angles of the two endpoints of the arc relative to the center of the circle, $\mathrm{size}\left(L_{i}\right)$ refers to the length of the arc $L_{i}$.

#### 2.3.2. Arc Fitting

On the basis of Section 2.3.1, the arcs of each quadrant are spliced sequentially, and if one of the following conditions is met, it is not considered an arc of the same circle.

(7) $$\begin{array}{c} \mathrm{thes}=\min(5,\min\left(R_{1},R_{2}\right)\times 0.1) \end{array}$$

(8) $$\begin{array}{c} \left|X_{1}-X_{2}\right|>\mathrm{thes}\;\mathrm{or}\;\left|Y_{1}-Y_{2}\right|>\mathrm{thes}\;\mathrm{or}\;\left|R_{1}-R_{2}\right|>\mathrm{thes} \end{array}$$

(9) $$\begin{array}{c} \frac{\left[\mathrm{angle}1,\mathrm{angle}2\right]\cap \left[\mathrm{angle}3,\mathrm{angle}4\right]}{\left[\mathrm{angle}3,\mathrm{angle}4\right]}<0.8 \end{array}$$

Among them, $X_{1},X_{2},Y_{1},Y_{2},R_{1},R_{2}$ are the circle parameters fitted by the arcs at both ends in Section 2.3.1, and angle1,angle2 are the angular area of the stitched arc relative to the center of the circle. Note that this value should be recorded in an array with a size of 360 in the program, because it may be discontinuous. $\left[\mathrm{angle}3,\mathrm{angle}4\right]$ is the position of the arc to be spliced relative to the center of the circle.

#### 2.3.3. Candidate Circle Detection

The spliced arc is then fitted to the circle parameters [X, Y, R] using least squares, and the points on the arc are first verified, where one of the following conditions is met. It is considered as an error detection, otherwise it is a candidate circle:

(10) $$\begin{array}{c} {\mathrm{Dist}}_{i}\;=\;2{\left(Px-X\right)}^{2}+{\left(Py-Y\right)}^{2}-R \end{array}$$

(11) $$\begin{array}{c} \frac{\sum {(\mathrm{Dist}}_{i}<\min\left(R/50,2\right)}{\mathrm{size}(\mathrm{Dist})}<0.9 \end{array}$$

(12) $$\begin{array}{c} \frac{\sum {\mathrm{Dist}}_{i}}{\mathrm{size}(\mathrm{Dist})}>2 \end{array}$$

#### 2.3.4. Remove Duplicate Circles

According to the method of the Refs. [17,20], the following formula is used to express the overlap ratio of circles, and when the overlap ratio is greater than 0.8, they are considered to be the same circle, and the circle with better sampling results is retained.

(13) $$\begin{array}{c} \mathrm{Ratio}\left(\mathrm{Cd},\mathrm{Ct}\right)=\frac{\mathrm{area}(\mathrm{Cd})\cap \mathrm{area}(\mathrm{Ct})}{\mathrm{area}(\mathrm{Cd})\cup \mathrm{area}(\mathrm{Ct})} \end{array}$$

where area(Cd), area(Ct) refers to the area of the circles Cd and Ct, respectively, and Ratio(Cd,Ct) refers to the overlap ratio of the two circles.

#### 2.3.5. Find the True Circle

We sampled to select true circles in the set of candidate circles. The sampling point coordinates can be calculated using the following formula:

(14) $$\begin{array}{c} \mathrm{Theta}=2\times \mathrm{Pi}\times i\;\;\;\;\;\;\;(i\in 0,1,\ldots ,36) \end{array}$$

(15) $$\left\{\begin{array}{cc} \mathrm{cx}=\mathrm{round}(X+R\times \cos(\mathrm{Theta})) & \\ \mathrm{cy}=\mathrm{round}(Y+R\times \sin(\mathrm{Theta})) \end{array}\right.$$

where cx, cy represent the aspect and ordinate coordinates of the sampling point, i represents the ith sampling point, and x, y, and r refer to the circle parameter. Since noise will cause a part of the circle information to be lost, when the number of verifications is greater than 12 times or when it is formed by multisegment arcs, we consider the circle parameter to be a true circle.

## 3. Proposed Circle Detection Algorithm

This section shows the flow of our proposed circle detection algorithm. Our proposed circle detection algorithm can be described as follows:

Step 1. Input a picture and perform 5 × 5 Median filtering on it;
Step 2. Adaptive Canny edge extraction is used to obtain edge point information;
Step 3. The edge point information is contoured to remove redundant edge points. The thinned edge points are connected into curves;
Step 4. Direction filtering of curves is used to suppress noise;
Step 5. A circular arc that divides a curve into segments;
Step 6. The arc is divided into five quadrants according to the position relationship between the arc and the center of the circle;
Step 7. Least squares circle fitting of circular arcs is performed within five quadrants. The set of candidate circles is found;
Step 8. Remove duplicate circles from candidate circles;
Step 9. Find the true circle in the candidate circle and mark it.

## 4. Results

In this section, we compare the proposed algorithm with five other algorithms. The first one is the random circle detection algorithm (RCD) proposed by Chen et al. [9]. We set the number of loops to 500,000 times. The second is a curvature-assisted circle detection algorithm (CACD) proposed by Zhen et al. [16]. The code comes from the author’s open source sharing [21], and the third is a sub-pixel circle proposed by Wang [12]. We repeated the algorithm from the paper and set the number of loops to 100,000. The fourth is a circle detection algorithm for arc fitting proposed by Lu [22], where the code comes from the author’s open source sharing [23], and finally, our algorithm. In order to unify the standard, all the above algorithms were executed in MATLAB R2019b, and all run on the same computer, using Intel Core i5-12400F. For the objectivity and accuracy of the experiment, the following four indicators will be used to measure: Precision, Recall, F-measure and Time. Time refers to the average time from the input image to finding the true circle in each dataset. The calculation formula of Precision, Recall and F-measure is as follows:

(16) $$\begin{array}{c} \mathrm{Precision}=\sum \frac{\mathrm{TP}}{\mathrm{TP}+\mathrm{FP}} \end{array}$$

(17) $$\begin{array}{c} \mathrm{Recall}=\sum \frac{\mathrm{TP}}{\mathrm{TP}+\mathrm{FN}} \end{array}$$

(18) $$\begin{array}{c} F-\mathrm{measure}=\sum 2\times \frac{\mathrm{Precision}\times \mathrm{Recall}}{\mathrm{Precision}+\mathrm{Recall}} \end{array}$$

We refer to the verification method [15,17,18,22,24,25,26], and when the coincidence of the circle is not less than 0.8, it is considered that they are the same circle. Treat it as a true positive (TP); otherwise, this is a false positive (FP), and the underlying fact that it is not correctly identified is considered a false negative (FN). Equation (13) was used to define the overlap ratio between circle Cd and circle Ct.

The test images in this paper were mainly from two Internet public datasets:

Dataset GH. This complex world dataset from the Ref. [27] consists of 257 gray images of different real-world scenes, with blurred edges and large variations in radius making detection difficult. Occlusion also brings inconvenience to the measurement.

Dataset MY. This complex world dataset comes from the Ref. [27], which consists of 111 pictures, and compared to the dataset GH, all datasets of MY are colored and contain more circles and involve more cluttered backgrounds, and almost elliptical circles may appear due to the camera’s viewing angle, where we used the labels that the author used to labeled the images.

### 4.1. Noise Test

**Figure 4:**
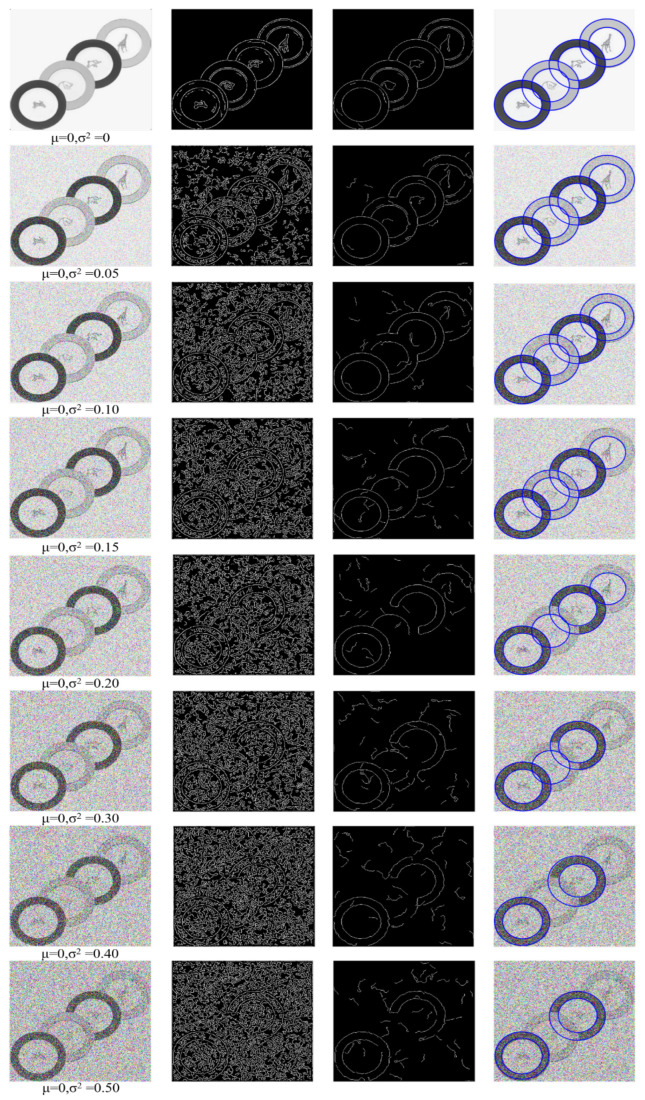
Results of our algorithm under different levels of Gaussian noise.

To test the performance of each algorithm under different levels of noise, we selected an image with a clear background and a circle consisting of strong edge points and weak edge points at the same time. The following Figure 4 shows the detection ability of our algorithm on images disturbed by Gaussian noise. The first image in each row represents different images disturbed by Gaussian noise with different variance. Additionally, the second image represents the result of edge extraction by adaptive canny algorithm. It can be seen from the results that the noise interferes with the boundary very seriously. The third picture is the intermediate result after we processed the picture. It can be seen that our algorithm reduces the noise as much as possible while retaining the arc. With interference, the fourth image is our circle detection result.

**Figure 5:**
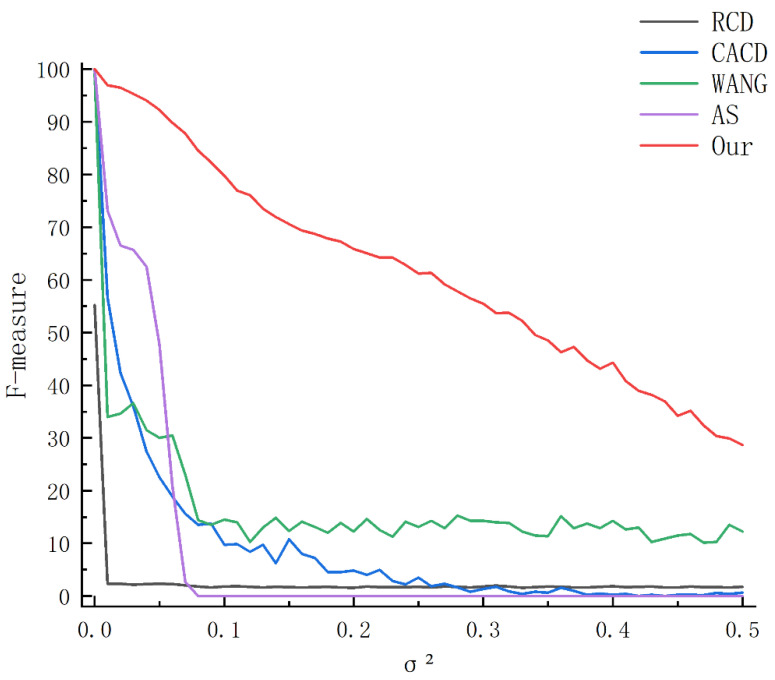
The F-measure results of five algorithms under different levels of Gaussian noise.

We describe the performance of different algorithms under Gaussian noise in Figure 5. Due to the randomness of Gaussian noise, we ran it 100 times with the same level of noise interference and show the results in Figure 4.

It can be seen from Figure 5 that both AS, CACD and our algorithm can detect all circles when there is no noise. When Gaussian noise just appears in the picture (variance is 0.01), the performance of the AS, WANG, CACD and RCD algorithms starts to reduce. Wang’s algorithm has an average F-measure of 0.9925 when there is no noise. When there is Gaussian noise in the image (variance of 0.01), the F-measure is reduced by about 66%, while the RCD algorithm is reduced by about 95%. This is because the noise Interference leads to false detections. When the variance of the AS algorithm is greater than 0.09, the F-measure is reduced to 0. When the variance of the CACD algorithm is greater than 0.08, the F-measure decreases to about 0.01 and remains relatively stable. The efficiency of our algorithm has the best performance on noisy images. When Gaussian noise first appeared on the image (variance 0.01), our F-measure remained at around 0.97, and as the noise increased, it still maintained a good performance.

### 4.2. Dataset Testing

**Figure 6:**
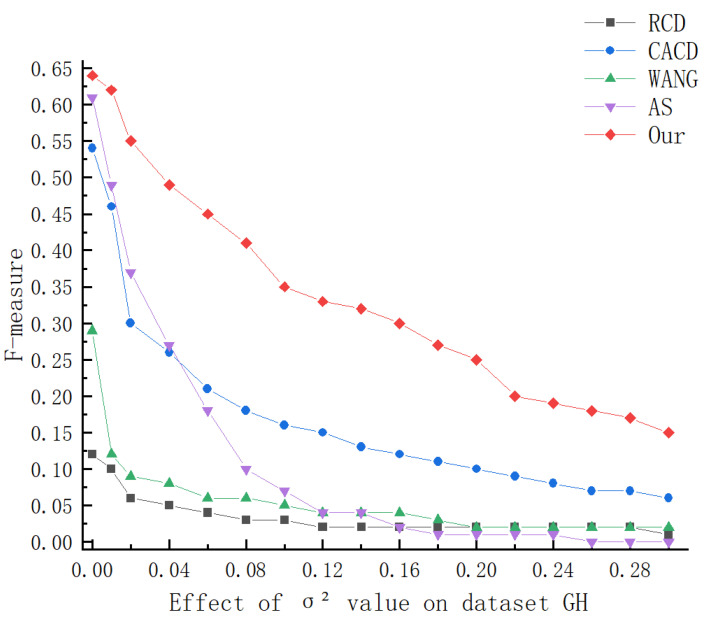
Variation of F-measure on dataset GH.

**Figure 7:**
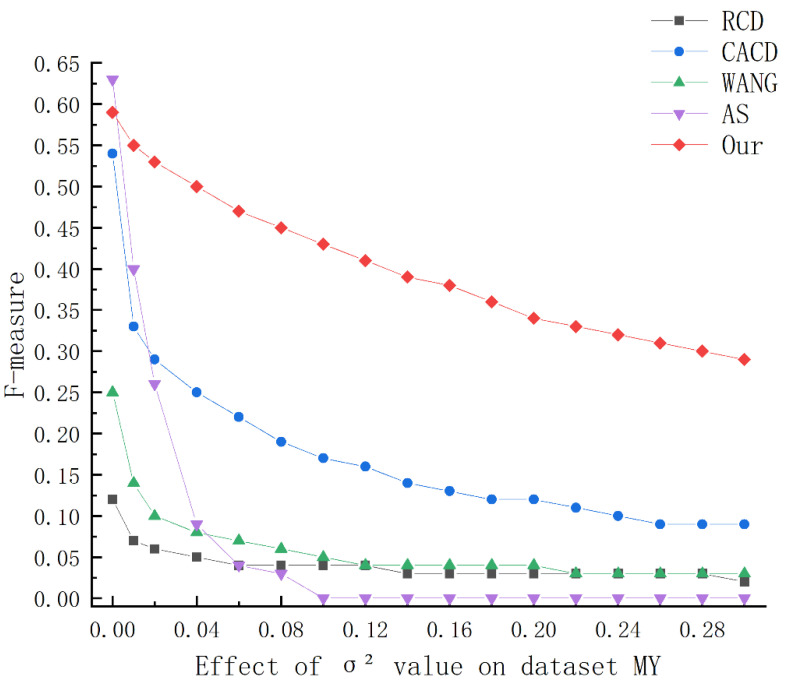
Variation of F-measure on dataset MY.

To test the stability of the algorithm, we selected two public datasets to test the algorithm. The following Figure 6 and Figure 7, and Table 1 and Table 2 show the average running results of the five algorithms under different levels of noise interference, and we marked the best results in bold. From the Table 1 and Table 2, it can be seen that:

The recall rates of RCD and Wang’s algorithms have always been stable, because the random circle detection algorithm will hardly be affected by noise when randomly selecting points to calculate circle parameters. However, with the increase in noise, their accuracy and F-measure decrease rapidly. The CACD and AS algorithms can maintain good performance without noise interference, but when the noise increases, the performance drops rapidly. When the variance is 0.04, their F-measures are reduced to 48% and 44% on GH and 46% and 14% on MY, respectively. Additionally, when the noise of the CACD algorithm increases, the running time of the program will also increase greatly. In contrast, the running time of the AS algorithm decreases as the noise increases, because the AS algorithm cannot detect arcs in the case of high noise. Our algorithm achieves the best results on both data sets. As the noise increases, other algorithms gradually lose their detection ability, while our algorithm can still maintain high performance.

The following Figure 6 and Figure 7 show the change of the F-measure in more detail. As the noise increases, the performance of all algorithms decreases to a certain extent. When the variance of Gaussian noise increases from 0 to 0.01, The performance of the Wang, CACD, and AS algorithms degrade more obviously. Additionally, although the AS algorithm has good performance in the case of no noise, its anti-noise performance is the worst. On the data set MY, the variance is higher than 0.1, and AS cannot detect any circle. The performance of the algorithms of RCD and WANG will quickly stabilize at a low level under noise interference. Although the anti-noise of CACD algorithm is better than AS, RCD, WANG, with the increase in noise, the time required for the program to run also increases rapidly. As can be seen from the above figures, with the increase in noise, the performance of our algorithm drops significantly slower than other algorithms, and the running time of the program is within 0.1 s.

## 5. Discussion

From the results in Section 4, it can be seen that our algorithm has the best performance under the influence of noise. This is mainly because we suppress the interference of noise through the randomness of noise. In our test, our algorithm can still detect the circle under the interference of Gaussian noise with a variance of 0.75. In the pre-processing stage, with the increase in noise level, our algorithm also needs more time to process noise information, but the algorithm time will be stable within 0.2 s. On the whole, this is acceptable. In the stage of circular arc fitting, the five-quadrant segmentation method we use also effectively reduces useless fitting, and saves verification time on the premise of ensuring high performance.

Based on our understanding of circle inspection, we will strive to apply this method to industrial practice areas in the future, such as inspection of PCB boards and Borehole detection.

## Figures and Tables

**Table 1:** Result on dataset GH.

Dataset	Index	RCD	CACD	Wang	AS	Our
Dataset GH	Precision	0.13	0.50	0.32	0.63	**0.65**
	Recall	0.57	**0.73**	0.51	0.69	0.71
$\mu =0,\sigma^{2}=0.00$	F-measure	0.12	0.54	0.29	0.61	**0.64**
	Time/s	1.68	1.07	1.55	0.1	**0.07**
Dataset GH	Precision	0.12	0.46	0.19	0.57	**0.66**
	Recall	0.57	0.57	0.49	0.51	**0.63**
$\mu =0,\sigma^{2}=0.01$	F-measure	0.1	0.46	0.12	0.49	**0.62**
	Time/s	1.68	1.14	1.95	**0.09**	0.11
Dataset GH	Precision	0.05	0.28	0.1	0.35	**0.58**
	Recall	**0.57**	0.32	0.47	0.25	0.48
$\mu =0,\sigma^{2}=0.04$	F-measure	0.05	0.26	0.08	0.27	**0.49**
	Time/s	1.69	2.30	1.91	**0.06**	0.14
Dataset GH	Precision	0.03	0.22	0.06	0.14	**0.51**
	Recall	**0.57**	0.22	0.47	0.09	0.38
$\mu =0,\sigma^{2}=0.08$	F-measure	0.03	0.18	0.06	0.1	**0.41**
	Time/s	1.69	2.78	2	**0.05**	0.16
Dataset GH	Precision	0.02	0.17	0.05	0.05	**0.41**
	Recall	**0.55**	0.19	0.47	0.04	0.3
$\mu =0,\sigma^{2}=0.12$	F-measure	0.02	0.15	0.04	0.04	**0.33**
	Time/s	1.71	2.99	2.1	**0.05**	0.16
Dataset GH	Precision	0.02	0.14	0.04	0.02	**0.36**
	Recall	**0.54**	0.16	0.47	0.02	0.27
$\mu =0,\sigma^{2}=0.16$	F-measure	0.02	0.12	0.04	0.02	**0.3**
	Time/s	1.71	3.14	2.18	**0.05**	0.17
Dataset GH	Precision	0.02	0.12	0.04	0.01	**0.31**
	Recall	**0.52**	0.14	0.46	0.01	0.22
$\mu =0,\sigma^{2}=0.20$	F-measure	0.02	0.10	0.02	0.01	**0.25**
	Time/s	1.73	3.24	2.25	**0.05**	0.17

**Table 2:** Result on dataset MY.

Dataset	Index	RCD	CACD	Wang	AS	Our
Dataset MY	Precision	0.17	0.62	0.35	**0.69**	0.63
	Recall	0.29	0.54	0.3	**0.65**	0.61
$\mu =0,\sigma^{2}=0.00$	F-measure	0.12	0.54	0.25	**0.63**	0.59
	Time/s	1.69	1.3	1.54	0.09	**0.08**
Dataset MY	Precision	0.15	0.45	0.2	0.55	**0.63**
	Recall	0.29	0.31	0.27	0.37	**0.55**
$\mu =0,\sigma^{2}=0.01$	F-measure	0.07	0.33	0.14	0.4	**0.55**
	Time/s	1.71	2.27	1.59	**0.09**	0.1
Dataset MY	Precision	0.08	0.36	0.11	0.17	**0.6**
	Recall	0.29	0.23	0.27	0.07	**0.49**
$\mu =0,\sigma^{2}=0.04$	F-measure	0.05	0.25	0.08	0.09	**0.5**
	Time/s	1.73	2.88	1.65	**0.07**	0.12
Dataset MY	Precision	0.05	0.29	0.08	0.06	**0.56**
	Recall	0.28	0.18	0.27	0.02	**0.44**
$\mu =0,\sigma^{2}=0.08$	F-measure	0.04	0.19	0.06	0.03	**0.45**
	Time/s	1.75	3.44	1.67	**0.06**	0.14
Dataset MY	Precision	0.04	0.24	0.06	0.00	**0.52**
	Recall	0.27	0.15	0.27	0.00	**0.39**
$\mu =0,\sigma^{2}=0.12$	F-measure	0.04	0.16	0.04	0.00	**0.41**
	Time/s	1.75	3.76	1.7	**0.05**	0.15
Dataset MY	Precision	0.03	0.2	0.05	0.00	**0.48**
	Recall	0.27	0.13	0.27	0.00	**0.36**
$\mu =0,\sigma^{2}=0.16$	F-measure	0.03	0.13	0.04	0.00	**0.38**
	Time/s	1.76	4	1.71	**0.05**	0.16
Dataset MY	Precision	0.02	0.18	0.05	0.00	**0.44**
	Recall	0.25	0.11	0.27	0.00	**0.32**
$\mu =0,\sigma^{2}=0.20$	F-measure	0.03	0.12	0.04	0.00	**0.34**
	Time/s	1.76	4.16	1.75	**0.05**	0.17

## Data Availability

https://github.com/zikai1/CircleDetection (accessed on 14 April 2022).
